# Multiple Human-Behaviour Indicators for Predicting Lung Cancer Mortality with Support Vector Machine

**DOI:** 10.1038/s41598-018-34945-z

**Published:** 2018-11-09

**Authors:** Du Ni, Zhi Xiao, Bo Zhong, Xiaodong Feng

**Affiliations:** 1 School of Economics and Business Administration, Chongqing University, Chongqing, 400044 PR China; 2 College of Mathematics and Statistics, Chongqing University, Chongqing, 400044 PR China

## Abstract

Lung cancer is still one of the most common causes of death around the world, while there is overwhelming evidence that the environment and lifestyle factors are predominant causes of most sporadic cancers. However, when applying human-behaviour indicators to the prediction of cancer mortality (CM), we are often caught in a dilemma with inadequate sample size. Thus, this study extracted 30 human-behaviour indicators of seven categories (air pollution, tobacco smoking & alcohol consumption, socioeconomic status, food structure, working culture, medical level, and demographic structure) from *Organization for Economic Cooperation and Development Database* and *World Health Organization Mortality Database* for 13 countries (1998–2013), and employed Support Vector Machine (SVM) to examine the weights of 30 indicators across the 13 countries and the power for predicting lung CM for the years between 2014–2016. The weights of different human-behaviour indicators indicate that every country has its own lung cancer killers, that is, the human-behaviour indicators are country specific; Moreover, SVM has an excellent power in predicting their lung CM. The average accuracy in prediction offered by SVM can be as high as 96.08% for the 13 countries tested between 2014 and 2016.

## Introduction

There is clear evidence for the reduction in lung cancer mortality (CM), especially in America and Europe^1^. However, lung cancer is still one of the most common causes of death around the world, and killed about 1.5 million people worldwide only in 2012. Lung CM has been the central issue of many epidemiological investigations, for it is of great value in defining the priorities of prevention, management, and treatment if we could precisely predict in advance. According to *Global Burden of Disease Study 2020*, the five-year survival of lung cancer (17.8%) will still be the lowest among all types of cancers^2^. So, exploring and predicting lung CM a few years earlier remain urgent.

Even though cancer is mainly a disease of genes, there is overwhelming evidence that the environment and lifestyle factors are predominant causes of somatic genetic alterations that lead to most sporadic cancers^3^ and there is also considerable variance in environment and lifestyle trends across the world in different countries. The variance would make the results of prediction imprecise, unstable and unrepresentative with any single input factor. Additionally, the data quality is often limited by small sample size, imperfect technology, the differences in clinical trials, the diversity of patient cohorts and health care system models. For example, recent studies indicated that there was a decreasing trend of lung CM in Western, Southern and Eastern European countries by applying tobacco control, yet the results were constrained to Asian countries even with a stable tobacco level^1^. This phenomenon may be caused by different trends of all the other kinds of human behaviours among those Asian citizens in different countries. As stated in the several other studies for prediction, it was often noted that a method that demonstrated the best performance for a given cohort might not perform as well as another method in another cohort^4^. Indeed, a single factor cannot always justify all the differences. For instance, air quality has been proved to be a strong factor to lung CM for the air being a complex mixture containing some known carcinogens, especially with long-term exposure^5–7^. If we just consider the effects of air quality in predicting, which is linear, another important cause of lung cancer, smoking, also needs our concern. If added, it will make the predicting model nonlinear and more complicated. In fact, many cohort studies have shown that diet^3^, aging structure^8^, socioeconomic level^2^, medical level^9^ were important factors contributing to CM. Also, there may be some cultural factors which might not be directly relevant, but substantially effective in a predicting model; for instance, working culture in Eastern Europe was much heavier than that in the western part, which caused the participants in Eastern Europe lacking time for exercising^10,11^. If we lose consideration for any one of these factors, the results thus obtained could not become persuasive enough. Hence, it is crucial to picture the human-behaviour trends globally as a whole by taking both the temporal patterns and future trends into consideration for predicting lung CM in future.

## Literature Review

Previous cohort studies claimed that the increasing trends of lung CM went along with the rise of air pollution^5–7^, for the particulate matter (PM) we breathe into our lungs may lead to cancerization. In particular, PM smaller than 2.5 μm aerodynamic diameter (PM2.5) shared notorious reputation in these studies^12^. Other particulates like PM10^13^, sulphur oxides, nitrogen oxides^6^, non-methane volatile organic compounds^14^ and carbon monoxide^15^ in the air have also been found to be indicators for lung cancer in different cohort studies. In addition to the factors mentioned above, higher levels of smoking^16^, alcohol consumption, which might impair immunity and have more possibility for cross-infection, have been proved to play a predominant role in predicting lung CM^17^. Given that different countries may have different smoking trends, it seems possible that the variance across countries might change lung CM. Socioeconomic factors like unemployment, occupational working condition have also been conceptualized as various pathogenic loadings to different systems of medical care, and attending more exercise may partially strengthen the immune system that reduces individual level of suffering from cancer^2^.

Mortality increase is also driven by cancer diagnosed in older adults and minorities^8^. Many empirical studies have shown that working culture was also associated with health^10^, more extended working hours could reduce the leisure time and physical activities, which were inversely associated with all-cause mortality in both men and women in all age groups^11^. And, population changes (due to worldwide immigration waves) in these countries have also been included in the analysis of cancer mortality because immigrants might usually keep the working cultures or life habits of the countries from which they migrated^18^.

Considering all above, the cohort studies reviewed mostly focused on only one variable in one single country or several cities, presenting purely descriptive death cases, or depended on the comparison across countries within a single calendar year without any wider considerations. Worse still, the archive data of human behaviours for each indicator were always short in years, which may make the results pretty random. For instance, although PM has been recognized as an air quality indicator since 1998, and if only based on correlation analysis, the data sets of PM sometimes cannot even pass Pearson correlation test.

To sum up, there are at least four essential knowledge gaps yet to be further addressed. First, few studies have delineated the current association between lung CM and multiple human-behaviour indicators specific to a country, and fewer studies have examined the correlation between the current indicators and lung CM across different countries. Second, based on the consensus of the disparities in lung CM across countries found more than two decades ago^19^, few studies raised the attention to the way how human-behaviour indicators across countries would modulate the prediction of lung CM in future. Thirdly, human-behaviour data relevant to lung cancer were normally short in years, and no traditional tools could satisfy the data mining in this field. Finally, despite a substantive variability between lung cancer and lung cancer related data for some data sets, the variability for a specific year or for a certain country cannot be figured out by traditional statistical analyses. To fill in these gaps, we are to apply multiple human-behaviour indicators in machine learning (ML) methods to delineate the current associations and to make the prediction in future.

Actually, there have been some studies using ML methods in the studies on lung CM. Up to now, artificial neural networks (ANNs) and decision trees (DTs) in ML have been used in cancer detection and diagnosis for nearly 30 years^20–22^. In medical research, particularly, ML methods enjoyed a wide application ranging from detecting tumours via X-ray and CRT images^23,24^, then to the classification of malignancies from proteomic and genomic (microarray) assays^25–27^. According to the latest PubMed statistics, more than 7510 papers have been published on the subject of ML in cancer prediction. It is worth noting that a new trend in the last decade in the use of other supervised learning techniques, namely support vector machine (SVM), is increasing in cancer prediction and prognosis^26,28,29^. However, the majority of these papers are concerned with using ML methods to identify, classify, detect, or distinguish tumours and other malignancies. In other words, ML methods have been used primarily as an aid to cancer diagnosis and detection^30^, which means ML methods have never been used in the data mining of multiple human behaviours. Thus, this paper intends to apply SVM to the exploring of the human-behaviour indicators for a comprehensive prediction with lung CM as a case.

As we know, SVM is one of the most popular off-the-shelf modelling algorithms, and one of its most powerful features is the potency in fitting the model with different kernels. SVM kernels can be regarded as different remedies to automatically combine the existing features in forming a richer feature space. To be specific, SVM has the following advantages over the traditional approaches. First, the human-behaviour archive data are normally short in years but rich in varieties. The shortage in length for a data set can be compensated by the varieties in width. In this case, SVM based on Vapnik-Chervonenkis (VC) theory is best suited, since SVM is a mature approach to solving the issues of the multidimensional function estimation, pattern recognition and time prediction^18,31,32^. Second, SVM aims to solve function approximation and regression estimation by using kernel functions of a data set, rather than the data itself, which can allow us more freedom in taking more features into consideration without any rigorous prior tests. This advantage also makes SVM very effective, especially for the cases having a high-dimensional space for input. Third, as compared with a traditional linear model, SVM can explore the relationship in an abstract multiple-axis dimension by putting the data (x_m_,y_*m*_) inside a two-dimensional space for figuring out the correlation between input and output in X, Y axis. This property equips SVM with robustness against the multicollinearity whereas linear regression fares poorly^33–35^. Fourthly, SVM uses the points in a subset of the input data, called ‘support vectors’, and only the points within the margin of lines supported by these vectors are considered error equals zero. The margin based on a subset of the data makes SVM suitable for those cases of small samples (less than 30 years of scale).

Thus, the advantages of SVM over traditional approaches mentioned above might directly improve the accuracy in prediction. If so, hopefully, the prediction of temporal trends of lung CM to be carried out in this study would quantify mortality variation, identify high-risk indicators, and delineate the potentials for preventive strategies. The epidemiological findings yet to be yielded in this study could be supportive for the cancer prevention and possibly screening strategies for policy-makers. In particular, this study also indends to figure out the weights of the human-behaviour indicators across different countries that lead to the disparities of lung CM, and to examine the power of SVM in prediction of lung CM with multiple human-behaviour indicators.

## Data Sources

This study employed two data sets. One was *Organization for Economic Cooperation and Development Database* (OECD Database); the other was *World Health Organization Mortality Database* (WHO Mortality Database).

OECD Database was selected based on the following reasons: (1) their great advantages in national coverage and long-term availability; (2) the high-quality for OECD members with historical data, even before a country became a member of the organisation; and (3) the reliability for the data of medical registration, which have been applied in many cancer studies. For some countries, if the coverage of the population is incomplete, the corresponding mortality produced might be implausibly low. By 2005, around one-third of the world population had been covered by mortality statistics^9^.

While almost all the European and American countries have comprehensive death registration systems, most African and Asian countries (including such populous countries as Nigeria, India and Indonesia) do not. Thus, 13 OECD countries worldwide in total were selected for this study, which involved United Kingdom, Czech Republic, Denmark, Ireland, Hungary, Finland, Germany, Netherlands, Poland, Spain, Switzerland, France and Canada, because the 13 countries shared the most common human-behaviour indicators without long missing gaps. Besides, the time period between 1998 and 2012 was chosen for human-behaviour indicators of the 13 OECD countries, for air-polluted data had been available since 1998 and lung CM for most countries had not come out yet before 2012.

WHO Mortality Database presented lung CM for the 13 countries were selected. Their great advantages were national coverage and long-term availability in higher income settings, although not all data sets were of the same quality. The lung CM has been age-standardized to the 1960s to the Segi world standard population and expressed per 100,000 population. Only the mortality of WHO Mortality Database was used in this study because there were no substantial differences between incidence and mortality rates and trends, even in economically developed countries based on poor survival.

Thanks to the two data sets mentioned above, 30 human-behaviour indicators of seven categories were obtained as follows:

### Air pollution

The first category of indicators concerning air pollution mainly came from the following three sub-data-sets of OECD Database: (1) UNECE-EMEP emissions Database (May, 2017), which was the meeting results of the convention on long-range trans-boundary air pollution (LRTAP Convention); (2) National Inventory Submissions 2017 (May, 2017), which was the results of the United Nations Framework Convention on Climate Change (UNFCCC, CRF tables), and (3) replies to the OECD Questionnaire on the State of the Environment and Comments from the member countries received before late September, 2017.

This category contained six indicators concerning air pollution in terms of man-made emissions: (1) sulphur oxides (SOx), (2) nitrogen oxides (NOx), (3) particulate matter (PM2.5, PM10), (4) carbon monoxide (CO), and (5) volatile organic compounds (VOCs) in thousands of tons per year. We also included data with (6) proportion of the population (percentage) affected by PM2.5 in percentage from 1998 to 2013 as an air pollution indicator.

### Tobacco smoking & Alcohol consumption

Numerous previous studies have related tobacco smoking and alcohol consumption to lung CM, which were the two indicators of the 2^nd^ category for the 13 OECD countries. Since 1968, OECD had been carrying out continuous surveys on different governmental websites, and grams per capita by the surveys were used as the indicator to determine the tobacco consumption for the 13 OECD countries from 1998 to 2013 by focusing on the tobacco consumers whose age was between 15 and above. Alcohol consumption in liters per capita was obtained in the similar way as the indicator of alcohol consumption for the 13 OECD countries from 1998 to 2013.

### Socioeconomic status

There are five socioeconomic indicators in the 3^rd^ category for the 13 OECD countries: (1) gross national income (GNI); (2) gross domestic product (GDP) for each country; (3) the unemployment rate was also chosen among the other macroeconomic indicators as a proxy for the economic conditions and uncertainty; (4) constant purchasing power parity (PPPs), allowing us to estimate what the exchange rate was between two currencies for the purchasing power of the two countries’ currencies, which was often used with GDP and GNI as a tri-indicator for the socioeconomic status in previous studies; and (5) consumer price index (CPI), also a strong indicator to estimate citizens’ wealth of a country. All these five indicators covered the years from 1998 to 2013 for the 13 OECD countries.

### Food structure

Six indicators in the 4^th^ category were quantified relatively with the cross-country data on average food supply from OECD Database as the current status of the 13 countries’ food structure: (1) total protein supply; (2) fat supply in grams per capita per day; (3) total calories supply in kilocalories per capita per day; (4) vegetables, (5) fruits, and (6) sugar supply in kilos per capita per year. All these data were picked out yearly between 1998 and 2013.

### Working culture

A few studies have investigated the association between long working hours and health. Their results revealed that long working hours could increase lung CM. Here, we used the annual working hours of the year as the 5^th^ category of indicators for the 13 OECD countries. The definition of the working time used here was the total number of hours over a year divided by the average number of people in employment of that year. The data were intended to make a comparison of change trends over time. Part-time workers were included as well as full-time workers for this indicator.

### Medical level

To define medical level internationally is extremely complex for at least two reasons. First, when medical technology is stable, the number of cured lung cancer cases is almost entirely related to the nation’s population as a whole. Second, the gross economy has to be taken into consideration. Thus, medical level of different countries was quantified simultaneously by mainly three indicators: (1) the proportion of medical workers in total workers employed; (2) the proportion of GDP as an expenditure put into medical care; and (3) the number of radiation equipment in hospitals. These comprised the 6^th^ category of indicators for the 13 OECD countries.

### Demographic structure

The information of democratic structure was quantified in this study in terms of age structure and male-female ratio. The data were obtained from the Population and Vital Statistics data sets, including the components of changes in the population during one year and mid-year for the 13 OECD countries. Since young people are generally immune to cancer and elderly people are more vulnerabable to lung cancer, we quantified the age structure further in the form of the following four indicators: (1) proportion of people of 65 years and above; (2) 80 years and above in a country. Some researches also revealed that lung cancer would affect the male and female differently; (3) male to female ratio from 1998 to 2013 of the 13 OECD countries; (4) death cases in different sexes were also collected. We used the 65+ ratio, 80+ ratio and gender ratio as the three indicators for the final analysis, which were quantified in thousands of persons and as rates in per 100000; (5) the proportion of youth population, who were unlikely to have lung cancer; and (6) the population changing by immigration. These six indicators were taken as the 7^th^ category of indicators for the 13 OECD countries.

## Methods

**Figure 1:**
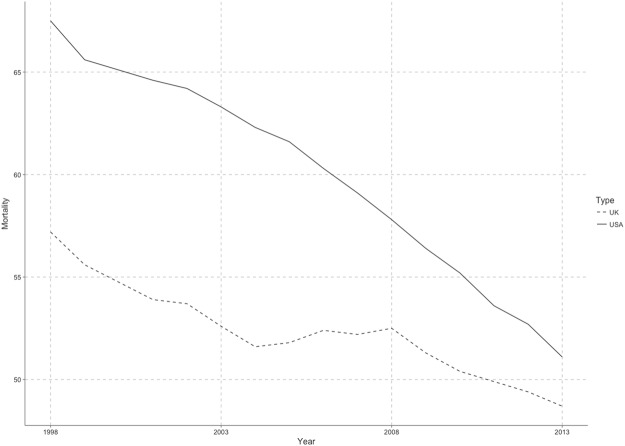
Lung CM (Deaths per 100,000 population, before being standardised) for U.S. and U.K (1998–2013). As we can see in Fig. 1, even though there are some fluctuations, both countries take on a decreasing trend of lung CM over the time after 1998. Because of the limited space, only the data for U.S. and U.K are shown for illustration.

There were two experiments in this study. Experiment 1 aimed to test the weights of each indicator across different countries, while Experiment 2 indended to examine the power for prediction in lung CM by recent 3 years. Before these two experiments were carried out, we would present a general view of lung CM for U.S. and U.K from 1998 to 2013 (Fig. 1) and make a brief introduction to the implementation of ML method for preparation. After we gathered all human-behaviour data from OECD Database, we replaced the missing data with the average values of their two neighbor years in order to improve the ML performance.

There were some specific steps for the implementation of ML method, which will be illustrated as follows:

- Step 1 We collected the data for human-behaviour indicators and split them into two sets, training set and evaluation set. All the data collected were standardized before being split and every data point should fall into the same parameter range. Otherwise, some data points might overwhelm the others, which would create more errors in analysis. Lung CM recorded for every country was from 30 to 80 people among 100000 population. Thus, we scaled all data into a range of [0, 100] by first subtracting their means and then dividing the results by the standard deviation.
- Step 2 We applied all the 30 indicators to look for the optimal parameters in SVM with the fitness function. Matlab (2016b) and LibSVM (version 1.7) were employed in this step. According to the annual data, the first 13 (1998–2010) data sets were used as training set, while the last 3 (2011–2013) as the evaluation set.
- Step 3 We obtained the predicting results with ML method.
- Step 4 We compared the results with those by the other predicting methods like Neuro-network (*NN*), and the traditional models like linear regression model for their predicting error rate, in order to justify the fitness of applying SVM in this study.

### Justification for applying SVM in this research

To justify the fitness of applying SVM in this study, we compared SVM with linear regression and NN to show the advantages of SVM over the traditional approaches in prediction.

#### Linear regression

Although linear regression like Logistic regression model is widely used in prediction, it has limitations if applied in this study. When considering the sample-feature ratio, we find there is no widely accepted answers to the issue at present. We used a rule of 20:1 (number of samples: number of features = 20:1) based on the suggestion made by a recent work^28^ for a more rigorous validation. In preparation process, in order to increase the validity of the data sets, we applied 30 indicators to guarantee the reliability of the outcome, which means we need at least 600 (30*20) outcomes. Unfortunately, we had only 16 years (from 1998 to 2013) in total, which was far less from being adequate, that is to say, the more features a data set has, the more likely the features are multi-collinear and the less reliable for interpreting the feature weights by coefficients. So, to predict with a linear regression is not suitable for this study.

#### Neuro-network

To compare the performance of SVM with NN, the linear SVM analysis was carried out for predicting lung CM with 30 predictors within 16 running years. To be specific, SVM regression calculation was done for forecasting lung CM by comparing the average error rate against that of NN performance for each country (Table 1).

**Table 1:** Error rates for NN and SVM in training process (Parameters of SVM have been cross-validated by Matlab Fitrsvm package).

Country	2011		2012		2013	
	NN	SVM	NN	SVM	NN	SVM
United Kingdom	1.24	0.70	1.36	1.70	1.92	2.01
Czech Republic	8.00	−0.05	8.99	0.35	11.88	2.41
Denmark	7.38	4.74	4.03	2.74	5.07	4.84
Ireland	6.24	4.90	3.13	2.90	5.15	5.00
Hungary	−5.46	1.44	−7.11	−0.30	−4.05	3.07
Finland	2.88	1.36	3.59	1.10	1.93	1.44
Germany	1.16	−0.01	1.37	−0.14	0.92	−0.01
Netherlands	0.22	−0.42	2.32	1.58	4.63	3.98
Spain	0.13	1.01	0.43	1.13	1.00	1.52
France	0.86	0.46	0.62	0.69	1.59	1.57
Canada	4.15	1.47	3.77	1.01	4.66	0.60
United States	3.96	2.42	4.66	2.16	5.44	3.33
Switzerland	−0.45	−1.03	1.08	0.77	0.97	0.76
Mean Square Error	NN	19.10		SVM	4.99	

The mean square error in training process for NN is as high as 19.10, while that offered by SVM is much smaller, only 4.99 on average. Therefore, the power of SVM in prediction remains much stronger than the traditional approaches like linear regression or NN do.

After preparation, there were seven steps involved in Experiment 1: (1) to gain trained SVM, named m1 here, from training samples with the true values of the 30 human-behaviour indicators (from 1998–2010) as the input and lung CM (from 1998–2010) as the output from the justification part; (2) to use the data of 2013 as the sample data, and to change the first indicator of the year 2013 (percentage of population exposed to more than 10 micrograms/m^3^) by adding the standardised value (the minimum and maximum value going between 0 and 100) by 10, while the values of the other 29 indicators were kept unchanged. Then, we got the new simulated data sets of the first indicator; (3) to put the new simulated data sets into trained SVM m1 to get the predicted lung CM ${\hat{{\rm{Y}}}}_{{\rm{i}}}$; (4) to subtract the simulated predicted lung CM ${\hat{{\boldsymbol{Y}}}}_{{\boldsymbol{i}}}$ by the absolute values of the original predicted lung CM ***Y***_***i***_ of the year 2013 as its identified weight for the first indicator; (6) to repeat the above-mentioned steps for the other 29 indicators; and (7) to draw the weight plot for all the 30 indicators of each country. In the end, we tested the differences between each indicator by Friedman test in SPSS 22.

In Experiment 2, we employed the human-behaviour indicators of the recent 3 years (2014–2016) to extend the annual data for further prediction of the three years after 2013. By so doing, we could predict the lung CM for each country up through the year of 2016, which has not been released by OECD Database yet (normally till 2013).

### Statistical Analyses

#### SVM

We employed Fitrsvm package of Matlab (2016b) for statistical analysis with SVM. The kernel is Gaussian kernel. Since Fitrsvm could find its own best-fit hyper-parameters automatically by minimizing five-fold cross-validation within a training set, we did not use any specifically settled parameters for Fitrsvm. For reproducibility, we used the random seeds with the ‘expected-improvement-plus’ function by Fitrsvm. Therefore, each country analysed in this study might have its own best-fit parameters automatically. Taking U.S as an example, the parameters settled for U.S model were as follows: The BoxConstraint (C) was settled at 0.0010113, the KernelScale (K) at 118.12, and the Epsilon (***ε***) at 3.2939.

#### NN

We used Back Propagation Neural Network, also a package of Matlab (2016b) for NN. For statistical analysis with NN, the ‘hiddenLayerSIze’ was set at 10.

#### MSE

The Mean Square Error (MSE) in Table 2 for comparing the power in prediction of NN with SVM was obtained through the following formula:

$${\rm{MSE}}=\,\frac{1}{{\rm{n}}}\sum _{{\rm{i}}=1}^{{\rm{n}}}{({{\rm{Y}}}_{{\rm{i}}}-{\hat{{\rm{Y}}}}_{{\rm{i}}})}^{2}$$

Where,Y_i_ is the true value of the lung CM; ${\hat{{\rm{Y}}}}_{{\rm{i}}}$ is the predicted lung CM value from both models.

**Table 2:** Prediction accuracy for the 12 OECD countries.

Country	Year	True Value	Predicted Value	Accuracy
United States	2014	49.8	51.01	97.57%
	2015	48	50.16	95.50%
United Kingdom	2014	48.2	49.22	97.88%
	2015	47	48.76	96.26%
Czech Republic	2014	42.2	45.88	91.28%
	2015	41.5	45.91	89.37%
	2016	41.2	45.62	89.27%
Denmark	2014	55.9	60.53	91.72%
	2015	54.3	60.54	88.51%
Ireland	2014	47.9	49.2	97.29%
Hungary	2014	73.9	74.22	99.57%
	2015	73.1	75.7	96.44%
	2016	73.4	74.7	98.23%
Finland	2014	30.7	32.03	95.67%
	2015	31.1	31.93	97.33%
Germany	2014	41	41.1	99.76%
	2015	40.6	40.75	99.63%
Netherlands	2014	54.2	54.87	98.76%
	2015	52.2	55.25	94.16%
	2016	52.4	51.2	97.71%
Spain	2014	38.7	40.16	96.23%
	2015	38.7	40.36	95.71%
France	2014	41.4	41.42	99.95%
Switzerland	2014	34.4	33.84	98.37%
	2015	34.1	34.05	99.85%
Average Value				96.08%

Only three countries have provided all the true values (Czech Republic, Hungary, and Netherlands) for recent three years. We can only display the data available and compare with our prediction.

#### Weight

The weight in Experiment 1 was the dynamic variation of lung CM when one input indicator varied. The weight of each human behaviour might change across countries, or for different time periods. Its value was assigned by the difference between the predicted value (after being added by 10 standardised value) and the original predicted value of year 2013 based on the evaluation data set of each human behaviour.

## Results

### Experiment 1

The design of Experiment 1 was derived from Sensitivity Analysis (SA), which is used to explore a mathematic model ${\rm{Y}}={\rm{f}}({X}_{1},\ldots ,{X}_{k})$, for how the output Y varies as the input *X*_*i*_ varies. The difference from SA involved that we used the trained SVM m1 instead of the other mathematic model, from the training set^36,37^. We set all the human-behaviour indicators to a certain level by changing the magnitude of a single feature, and in this way, we could check how lung CM would change after being added by 10 for each feature after being standardised. So, the relative importance and contribution of each indicator could be quantified with their corresponding changes in weights before and after being added by 10.

**Figure 2:**
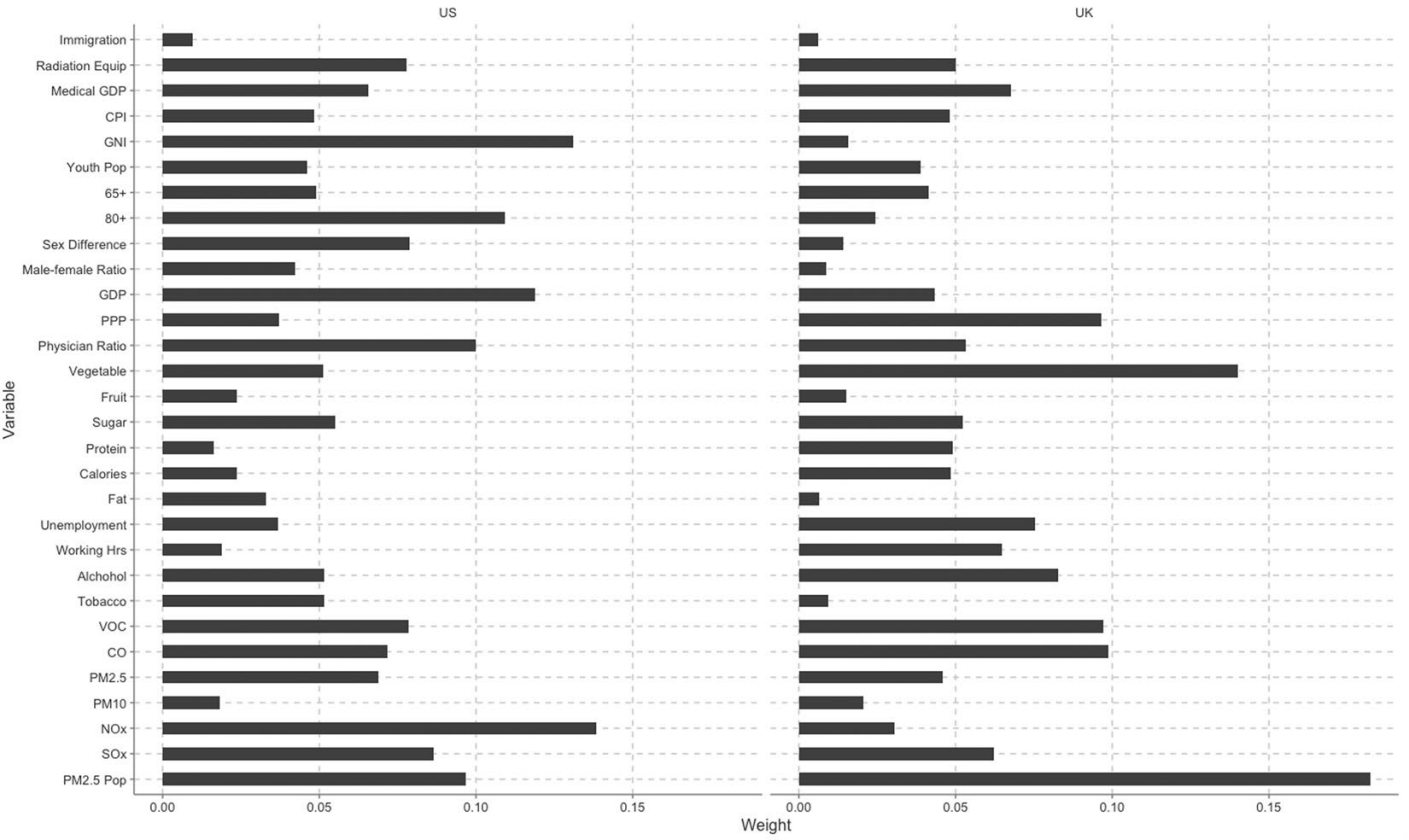
Weights of different human-behaviour indicators for U.S. and U.K. The top 3 indicators affecting population in U. S. are NOx, GNI, and GDP, while PM2.5, vegetable supply, and CO emission in U. K. Also, we can see that different countries are affected by different key human-behaviour indicators.

We did the Pearson correlation with the variables from the UK and US. As can be seen in Fig. 2, the United States kept the other indicators stable, while its NOx (Pearson coefficient of correlation, r = 0.98, *p* < 0.001) took the first place in lung CM. GNI ranked the second, which was negatively correlated with lung CM over time (r = −0.96, *p* < 0.001) and GDP was negatively correlated with gender inequality (r = −0.97, *p* < 0.001). Those variables such as NOx, GPD supply and GNI are new to us in addition to the common causes like tobacco smoking and air pollution. Unlike U.S, UK had its PM2.5 as the most serious killer (r = 0.98, *p* < 0.05), and the vegetable supply ranked the second (r = −0.63, *p *< 0.01), and CO emission did the third (r = 0.95, *p* < 0.001). Thus, the results obtained in this study may provide governments and epidemiological researchers with a new approach to identifying new variables for controlling lung CM at a macro level for a specific country.

Next, a non-parametric test was used to assess the differences between groups. To be specific, Friedman test was carried out to check the differences of each human- behaviour indicator with SPSS 22.0. As the statistical results ${{\rm{\chi }}}_{(30)}^{2}=47.526$, *p* = 0.006 indicated, different types of human-behaviour indicators play significantly different role in predicting lung CM.

### Experiment 2

In Experiment 2, the average changing rates of the last 16 years before 2013 (1998–2013) were employed to expand our time length for the prediction of the future three years after 2013. By so doing, the lung CM for each country up to the year 2016 could be predicated.

As we know, not all countries will upload their lung CM data in time. This may result in a time lag of human-behaviour indicators in OECD Database. In this case, we could compare the predication for year 2014–2016 obtained in this study with the real archive that has been uploaded into OECD Database already. Till the day we finished our experiment, only eight out of the 13 countries had released their lung CM for year 2014 and three for 2015.

**Figure 3:**
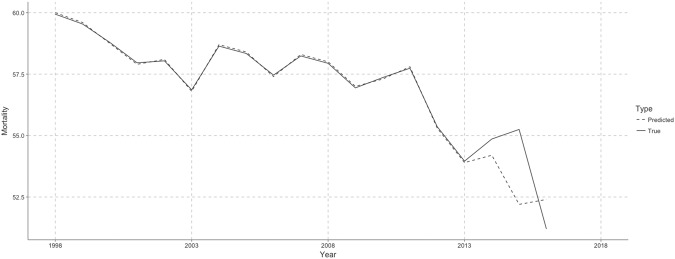
Lung CM (Deaths per 100,000 population) Prediction for Netherlands in 2014, 2015 and 2016. Here, we only display the data of Netherlands as an example for the other countries for contemporary trends of a decline in lung CM because of the delayed data. 1998–2013 are training set. 2014–2016 are predictions for the recent three years.

The process for statistical analysis in Experiment 2 was similar to what had been done in Experiment 1, that is, we first integrated the data sets of 2014, 2015 and 2016 with the current changing rate into the original data sets, which had been standardized with the range of [0,100] before. Then they were split into training set and evaluation set, that is, one from 1998 to 2013 for training, and the other 3 years from 2014 to 2016 for evaluating the prediction. The comparison of the true and predicted results are shown in the Fig. 3 below.

As indicated in Table 2, 12 countries (except Canada) announced their lung CM. By comparing the results announced with those predicated in Table 2, we could know that the average prediction accuracy on average is up to 96.08%, which proves an excellent performance made by SVM in approximating lung CM in application.

## Conclusions

The results of Experiment 1 suggested that the fluctuations of lung CM in different countries were caused by different human-behaviour indicators, indicating that a causal direction from shifts in daily human behaviours to those in lung CM did exist. The correlation is not so robust, but still changing if the other human-behaviour dimensions are simultaneously controlled. Based on the changes of the ranks for the 13 OECD counties, to think further, it is shown by the results yielded in this study that this effect may not be confined to the changes only inside the 13 OECD countries. In fact, every country outside the 13 OECD countries might have its own way being affected by the combinations of human-behaviour trends.

The results of Experiment 2 proved that lung CM in the 13 OECD countries could be predicted with multiple human-behaviour indicators. The accuracy for these countries that have released the lung CM was all above 90%, which revealed an excellent power of SVM in prediction, and taking German with the least made-up data as an example, the accuracy in prediction for German was as high as up to 99.76% in 2014 and 99.95% in 2015.

Taken together, these findings claimed a novel way for predicting how lung cancer might kill people in different countries—by applying multiple human-behaviour indicators with ML methods, a blind site to traditional approaches in prediction for cohort studies, since the traditional approaches in prediction could neither fully justify the differences from a broader view, nor thoroughly and effectively integrate those factors that may cause errors and diminish the power in prediction.

Before concluding, we should note that there are at least several limitations of this study. First, the cancer mortality from WHO mortality database are prone to a lower validity than the registry cancer statistics, mainly because of the difficulties in ascertaining and certifying the death causes. However, short-term rather than long-term predictions based on these databases have been proved to be satisfactorily robust and could still provide a reasonable picture of the current burden in these countries involved^32^, and long-term prediction with a higher accuracy for lung CM could be done with more valid registered cancer statistics for further studies in future. Second, only 30 human-behaviour indicators from the same data set could not definitely ensure the forecasting results concerning lung CM^31^. More indicators apparently not directly related, like education attainment, which should have been included in this study, could be taken into consideration in future. Third, we only highlighted the power of SVM in prediction in this article, and neglected the coefficients caused by the multi-linearity effects among the 30 indicators. Moreover, although there are indications of significant correlation between these human-behaviour indicators and lung CM, further approaches involving experiments and agent-based modeling of societal changes via computer-aided simulation could help further corroborate causal inferences. Admittedly, the present study has systematically presented ecologically-derived explanations for the forces that may cause societies to change regarding levels of lung CM, further detailed researches should and could surely allow more room for the other effective indicators.

Beyond theoretical implications, these findings do have practical implications to policymakers. Our results suggested that efforts to better human behaviours, such as less overwork, more investment in free medical care, more active physicians, better food structure and reduced tobacco and alcohol supply at the same time, might also benefit lung cancer prevention. What is more, since the figures of official cancer mortality generally become available with a few years’ lag, ML like SVM is really a practical tool for predicting mortality with the other available data.

## Data Availability

The data sets generated during and/or analysed in the current study are available from the corresponding author on reasonable request.
